# Glymphatic system dysfunction in patients with cluster headache

**DOI:** 10.1002/brb3.2631

**Published:** 2022-05-17

**Authors:** Jinseung Kim, Dong Ah Lee, Ho‐Joon Lee, Bong Soo Park, Junghae Ko, Si Hyung Park, Yoo Jin Lee, Il Hwan Kim, Jin Han Park, Kang Min Park

**Affiliations:** ^1^ Department of Family Medicine Busan Paik Hospital Inje University College of Medicine Busan Korea; ^2^ Department of Neurology Haeundae Paik Hospital Inje University College of Medicine Busan Korea; ^3^ Department of Radiology Haeundae Paik Hospital Inje University College of Medicine Busan Korea; ^4^ Department of Internal medicine Haeundae Paik Hospital Inje University College of Medicine Busan Korea

**Keywords:** cluster headache, diffusion tensor imaging, glymphatic system

## Abstract

**Introduction:**

The aim of this study was to investigate alterations of the glymphatic system function in patients with cluster headache.

**Methods:**

We enrolled patients with cluster headache and healthy controls, and they underwent brain magnetic resonance imaging (MRI), including diffusion tensor imaging (DTI). We used the MRIcron and DSI studio programs for DTI preprocessing and DTI analysis with perivascular space (DTI‐ALPS) index calculation.

**Results:**

Fourteen patients with cluster headache and 23 healthy controls were enrolled. The DTI‐ALPS indexes of the groups were significantly different. The DTI‐ALPS index for the patients with cluster headache was lower than that for the healthy controls (1.586 vs. 1.786, *p *= 0.044). There was a significant negative correlation between the DTI‐ALPS index and age in the patients with cluster headache (*r* = −0.549, *p *= 0.042). However, the DTI‐ALPS index was not associated with other clinical characteristics, including disease duration and headache intensity (*r* = −0.405, *p *= 0.150; *r* = −0.048, *p *= 0.869, respectively).

**Conclusion:**

Patients with cluster headache had a lower DTI‐ALPS index than the healthy controls; this might indicate glymphatic system dysfunction in the patients with cluster headache. Further research is required to determine whether glymphatic system dysfunction is related to the pathophysiology of cluster headache.

## Graphical abstract

This study investigated the alterations of the glymphatic system function associated with cluster headaches. Patients with cluster headaches had a lower DTI‐ALPS index than the healthy controls. It provides insights for further research into the association between glymphatic system dysfunction and cluster headaches and the evidence of this association will guide interventions for cluster headaches and improve patient outcomes.

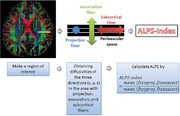

## 1 INTRODUCTION

Until the glymphatic system theory was published in 2012, it was assumed that the brain, like any other organ, recycled all of its protein waste (Rubinsztein, 2006). However, the concept has changed since the introduction of the glymphatic system. The glymphatic system is now thought to be a brain‐wide perivascular pathway for the exchange of cerebrospinal fluid (CSF) and interstitial fluid (ISF) in the central nervous system, promoting effective elimination of solutes and waste products from the brain (Iliff et al., 2013). Alzheimer's dementia, aging, traumatic brain injury, brain hemorrhage, cerebrovascular disease, Parkinson's disease, and normal pressure hydrocephalus (NPH) are all known to be linked to glymphatic system dysfunction (Gaberel et al., 2014; Goulay et al., 2017; Iliff et al., 2014; Jiang et al., 2017; Kress et al., 2014; Mestre et al., 2018; Peng et al., 2016; Plog et al., 2015; Wang et al., 2017).

The use of magnetic resonance imaging (MRI) to visualize glymphatic system function is critical for understanding the pathophysiology of various neurodegenerative disorders, and it can be a therapeutic target or be used to determine therapeutic efficacy. MRI has been used to visualize glymphatic system function in several ways, and the use of intrathecal gadolinium‐based contrast agents (GBCAs) is the most representative. There have been reports of studies using MRI and patients with NPH, and the intracranial kinetics of GBCAs significantly differ between patients with NPH and controls (Edeklev et al., 2019; Ringstad et al., 2018). However, the method involving the use of intrathecal GBCAs is invasive, and the degree and speed of penetration vary from person to person (Ringstad et al., 2018). As a result, the application of intrathecal GBCAs to investigating glymphatic system function has been challenging and wrought with several limitations.

Diffusion tension imaging (DTI) has been recently utilized to assess glymphatic system function in several studies (Debaker et al., 2020; Yokota et al., 2019). DTI analysis with the perivascular space (DTI‐ALPS) calculation method is based on the idea that perivascular ISF movement in the white matter near the lateral ventricles is prominent along the parallel medullary veins (Yokota et al., 2019). The DTI‐ALPS method requires no GBCAs, uses clinically common DTI data, and can be performed retrospectively with the use of previous imaging data; therefore, it is highly useful for research.

Cluster headache is the most severe types of headaches, and they are characterized by ipsilateral pain localized to the orbital, supraorbital, and/or temporal areas, as well as associated autonomic processes. Lacrimation (tearing), conjunctiva injection (sclera redness), rhinorrhea, nasal congestion, hyperhidrosis (excessive sweating), and eyelid edema occur ipsilateral to the pain, with only 3% of individuals showing no autonomic symptoms (Sjaastad, 1992). They last for 15−80 min and occur up to eight times a day (Society, 2013). Sleep has been identified as a common trigger for cluster headache. A previous questionnaire‐based study involving 275 patients with cluster headache showed that 80% of patients reported that sleep is a trigger for their headache episodes (Barloese, Lund et al., 2015). Patients with cluster headache scored higher on the Pittsburgh Sleep Quality Index than healthy people, indicating a low self‐perception of sleep quality. In addition, patients who report that sleep is a trigger for their headache attacks scored higher than those who report sleep is not a trigger factor (Barloese, Lund et al., 2015). Furthermore, the majority of headaches occur at night during sleep, and the greatest issue for patients with cluster headache is sleep inefficiency (Jensen et al., 2007). Cognitive dysfunction does not last beyond headaches, but patients with cluster headache usually experience repeated cognitive deterioration (Favoni et al., 2018). Patients with Alzheimer's dementia also have glymphatic system dysfunction (Nedergaard & Goldman, 2020; Taoka et al., 2017). Considering all these, and the evidence that the glymphatic system is activated especially during sleep, patients with cluster headache may have decreased glymphatic system function.

This study aimed to investigate alterations of the glymphatic system function in patients with cluster headache. The glymphatic system and cluster headache are both linked to sleep, and our research is based on the hypothesis that patients with cluster headache have glymphatic system dysfunction. To our knowledge, there have not been studies on this.

## 2 METHODS

### 2.1 Participant: Patients with cluster headache and healthy controls

**FIGURE 1:**
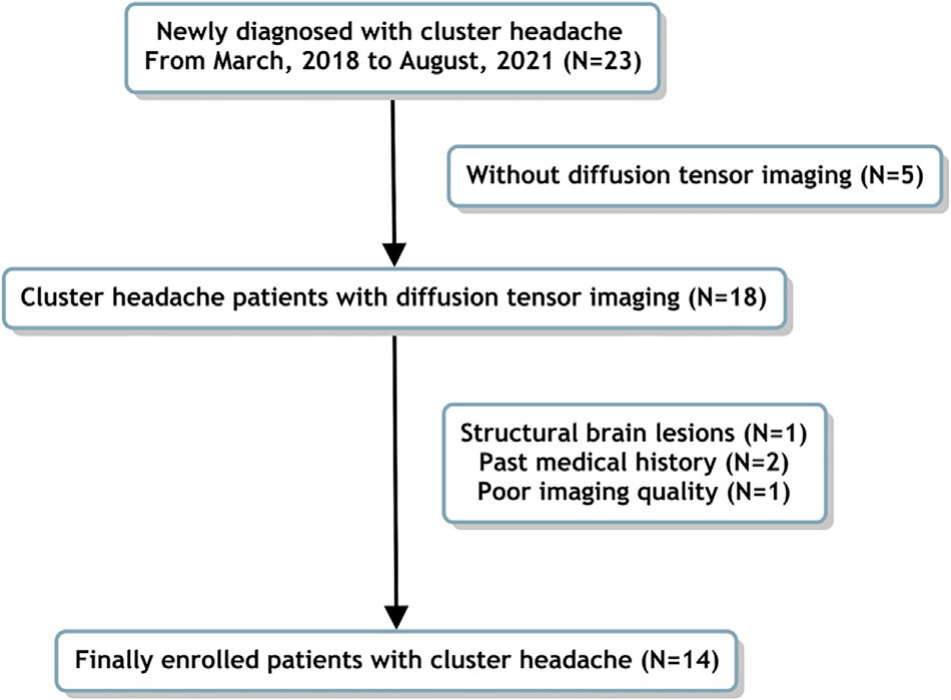
Selection process for the patients with cluster headache in this study

This study was conducted retrospectively in a single hospital and approved by the institutional review board at Haeundae Paik Hospital. We enrolled patients with cluster headache based on the third edition of the International Classification of Headache Disorders and the following criteria (Headache Classification Committee of the International Headache Society [IHS], 2018): (1) newly diagnosed episodic cluster headache at our hospital between March 2018 and August 2021, who were not taking any preventive medications for cluster headache. (2) Brain MRI including DTI. We excluded patients with the following: (1) previous history of any medical or neurological disease, except cluster headache, (2) structural lesion on brain MRI, or (3) having poor quality DTI for analysis. Figure 1 shows the selection process for patients with cluster headache in this study. We reviewed the medical records to obtain demographic and clinical characteristics, such as age, sex, disease duration (time interval from headache onset to MRI taken), headache intensity, and headache laterality (side with most headaches) for the patients with cluster headache.

We also enrolled age‐ and sex‐matched healthy controls who had no previous history of any medical or neurological disease. All healthy controls underwent brain MRI including DTI, and they had no structural lesions on brain MRI. They had been already recruited from our previous study, which had enrolled a total of 150 healthy participants (Jang et al., 2017). Of the 150 patients, those who did not consent to the use of their data in this study were excluded. Finally, we included the remained healthy participants in the control group with similar age and sex as the patients with cluster headache.

### 2.2 Diffusion tensor imaging acquisition

All MRI scans of the participants were obtained using the same MRI scanner, which was a 3 tesla with a 32‐channel head coil (AchievaTx; Phillips Healthcare). From March 2018, routine brain MRI protocols at our hospital for patients with cluster headache included DTI. All of the MRI scanning was conducted during out‐of‐out phase of headache attacks in patients with cluster headache, and MRI scans were performed during the daytime in all participants (before 5 pm). The participants underwent the same brain MRI protocol, which included three‐dimensional (3D) FLAIR imaging, coronal T2‐weighted imaging, 3D T1‐weighted imaging, and DTI. FLAIR and T2‐weighed imaging were used to evaluate the structural abnormalities of the brain. The detailed MR parameters for DTI with 32 different diffusion directions have been described in our previous studies (Lee et al., 2021)

### 2.3 Diffusion tensor imaging preprocessing and diffusion tensor image analysis along with the perivascular index calculation

**FIGURE 2:**
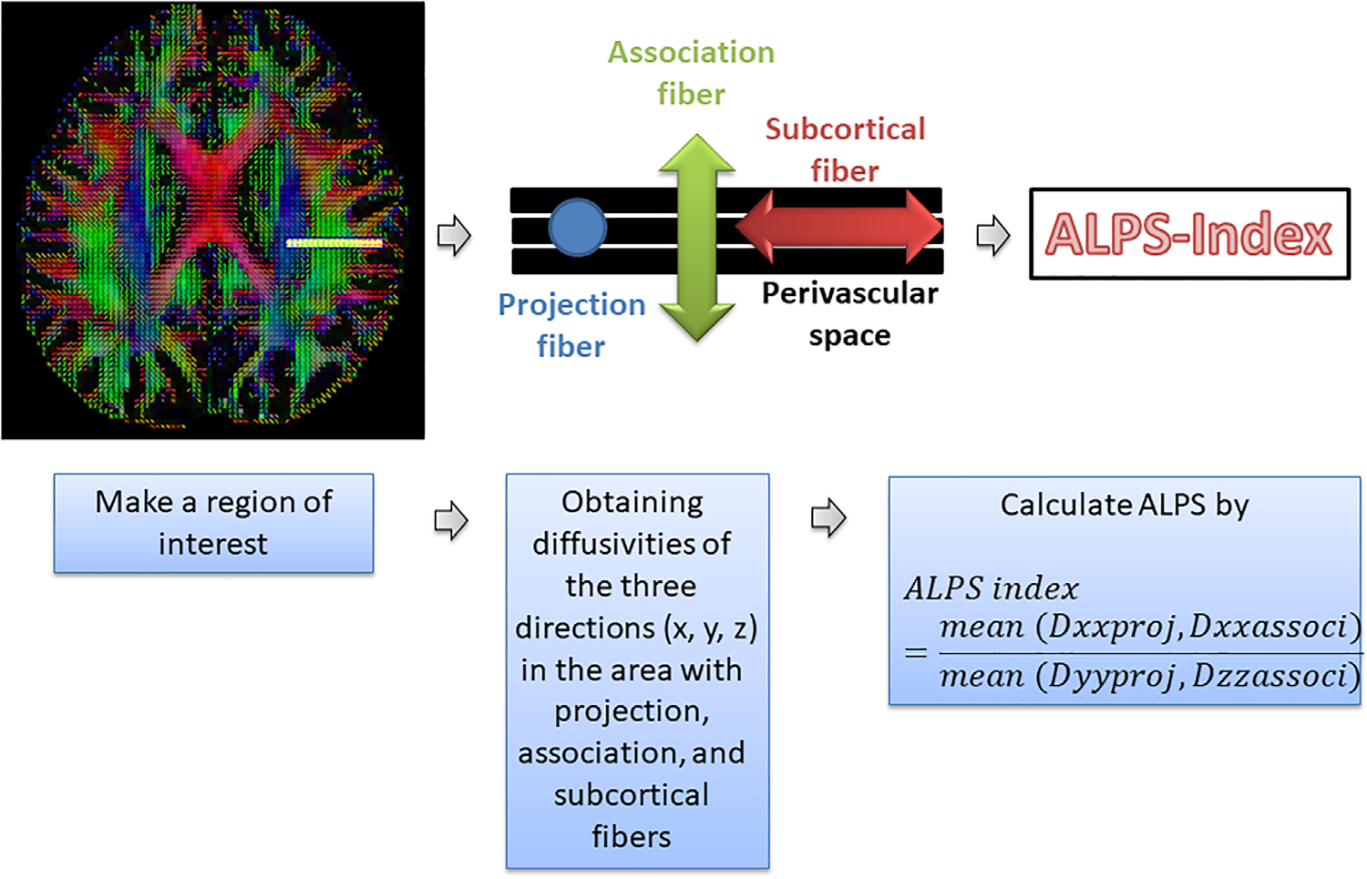
Diffusion tensor image analysis with the perivascular space (DTI‐ALPS) index calculation. The figure was generated from our previous study (Lee et al., 2021)

We used the MRIcron and DSI studio programs for DTI preprocessing and DTI‐ALPS index calculations. The steps for DTI preprocessing and DTI‐ALPS index calculation were described in our previous study (Lee et al., 2021). The preprocessing for DTI included setting up a mask, reconstruction with the DTI method, and fiber tracking. We, subsequently, calculated the DTI‐ALPS index (Figure 2) (Lee et al., 2021; Taoka et al., 2017). We drew a rectangular region of interest (ROI) and obtained the fiber orientation and diffusivities of the three directions along the *x*‐, *y*‐, and *z*‐axes as voxel levels at the ROI. Finally, the DTI‐ALPS index was obtained using the following formula (Taoka et al., 2017):

$$\mathrm{ALPS}\;\mathrm{index}=\frac{\mathrm{mean}\;\left(\mathrm{Dxxproj},\;\mathrm{Dxxassoci}\right)}{\mathrm{mean}\;\left(\mathrm{Dyyproj},\mathrm{Dzzassoci}\right)},$$

where Dxxproj is diffusivity along the *x*‐axis in the projection fiber, Dxxassoci is diffusivity along the *x*‐axis in the association fiber, Dyyproj is diffusivity along the *y*‐axis in the projection fiber, and Dzzassoci is diffusivity along the *z*‐axis in the association fiber.

### 2.4 Statistical analysis

A primary analysis of these data was conducted using an a priori test. No statistical power calculations were performed before the study. The sample size was based on the available data. Comparisons of the demographic characteristics of the patients with cluster headache and healthy controls were conducted using an independent sample *t*‐test or the chi‐squared test. Comparisons of the diffusivities and DTI‐ALPS indexes of the groups were performed using an independent sample *t*‐test. Correlation analysis was performed using Pearson's correlation coefficient. Categorical variables are presented as frequencies and percentages, whereas continuous variables are presented as mean ± standard deviation or median. Statistical significance was set at a two‐tailed *p *< 0.05. MedCalc® Statistical Software version 20.014 (MedCalc Software Ltd, Ostend, Belgium; https://www.medcalc.org) was used for the statistical analysis.

## 3 RESULTS

### 3.1 Demographic and clinical characteristics of the patients with cluster headache

We enrolled 14 patients with cluster headache and 23 healthy controls. Table 1 shows the demographic and clinical characteristics of patients with cluster headache. Their ages and sex were not different from those of the healthy controls (42.2 vs. 40.6 years, *p *= 0.696; 13/14 vs. 21/23, *p *= 1.000). Most of the patients with cluster headache were men.

**TABLE 1:** Demographic and clinical characteristics in patients with cluster headache and healthy controls

	Patients with cluster headache (N = 14)	Healthy controls (N = 23)	*p*‐Value
Age, years	42.2 ± 16.9	40.6 ± 8.5	0.696
Male, *n* (%)	13 (92.8)	21 (91.3)	1.000
Disease duration, years	2.1 ± 1.6		
Headache intensity, visual analog scale	8.7 ± 0.8		
Right side attack, *n* (%)	7 (50.0)		

### 3.2 Differences between the diffusivities and diffusion tensor image analyses along with the perivascular indexes of the patients with cluster headache and healthy controls

The diffusivities along the *x*‐, *y*‐, and *z*‐axis of the projection, association, and subcortical fibers of the patients with cluster headache and healthy controls were not statistically different (Table 2).

**TABLE 2:** Differences between the diffusivities and diffusion tensor image analysis with the perivascular space (DTI‐ALPS) index in patients with cluster headache and healthy controls

	Patients with cluster headache (N = 14)	Heathy controls (N = 23)	*p*‐Value
Projection fiber			
Dxx	0.602 ± 0.081	0.613 ± 0.099	0.715
Dyy	0.429 ± 0.133	0.381 ± 0.097	0.220
Dzz	1.034 ± 0.149	1.066 ± 0.142	0.518
Association fiber			
Dxx	0.598 ± 0.080	0.627 ± 0.111	0.410
Dyy	1.080 ± 0.121	1.166 ± 0.146	0.074
Dzz	0.347 ± 0.102	0.320 ± 0.065	0.336
Subcortical fiber			
Dxx	1.082 ± 0.163	1.144 ± 0.137	0.227
Dyy	0.620 ± 0.186	0.681 ± 0.196	0.362
Dzz	0.538 ± 0.197	0.649 ± 0.210	0.122
DTI‐ALPS index	1.586 ± 0.371	1.786 ± 0.212	*0.044

(×10^−3^ mm^2^/s).

Abbreviations: Dxx, diffusivity along the *x*‐axis; Dyy, diffusivity along the *y*‐axis; Dzz, diffusivity along the *z*‐axis; DTI‐ALPS index, diffusion tensor image analysis with the perivascular space index.

* *p *< 0.05.

The DTI‐ALPS indexes for the groups were significantly different. The DTI‐ALPS index for patients with cluster headache was lower than that for healthy controls (1.586 vs. 1.786, *p *= 0.044).

### 3.3 Correlation analysis of diffusion tensor image analysis with the perivascular index and demographic/clinical characteristics

**FIGURE 3:**
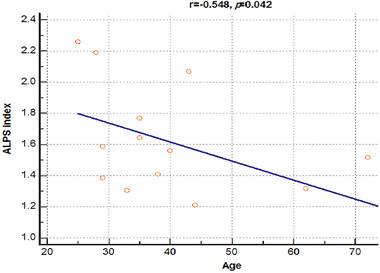
Correlation analysis of diffusion tensor image analysis with the perivascular space (DTI‐ALPS) index and age in patients with cluster headache. There was a significant negative correlation between the DTI‐ALPS index and age in patients with cluster headache

There was a significant negative correlation between the DTI‐ALPS index and age in patients with cluster headache (*r* = −0.549, *p *= 0.042) (Figure 3). However, the DTI‐ALPS index was not correlated with other clinical characteristics, including disease duration and headache intensity (*r* = −0.405, *p *= 0.150; *r* = −0.048, *p *= 0.869, respectively).

## 4 DISCUSSION

The following were the main findings of this study. (1) The DTI‐ALPS indexes of the patients with cluster headache and healthy controls were different. The DTI‐ALPS index was lower for patients with cluster headache than for healthy controls. (2) There was a significant negative correlation between the DTI‐ALPS index and age for the patients with cluster headache. (3) The DTI‐ALPS index was not correlated with other clinical characteristics such as disease duration or headache intensity for patients with cluster headache.

This was the first study to evaluate glymphatic system function in patients with cluster headache. The lower DTI‐ALPS index of patients with cluster headache than that of healthy controls in this study implies that patients with cluster headache have glymphatic system dysfunction. Glymphatic system dysfunction is important in the pathophysiology of neurovascular, neurodegenerative, neuro‐inflammatory diseases, and traumatic brain injury, as well as age‐related changes in brain functions (Sun et al., 2018). There are also reports of the potential role of the glymphatic system in headache disorders, especially migraine (Messlinger, 2018; Schain et al., 2017; Toriello et al., 2021; Vgontzas & Pavlovic, 2018). Cortical spreading depression, the neural event underlying migraine aura, results in a temporary impairment of glymphatic flow in a mouse model via paravascular space closure for several minutes after inducing cortical spreading depression, followed by a gradual recovery over half an hour (Schain et al., 2017; Toriello et al., 2021). In addition, calcitonin gene‐related peptide plays a critical role in the pathophysiology of headaches, particularly migraine. The calcitonin gene‐related peptide may be excreted in three compartments: venous blood plasma, cerebrospinal fluid, and possibly the lymphatic system. Thus, glymphatic system dysfunction may be associated with increased levels of calcitonin gene‐related peptide, which may produce a headache (Messlinger, 2018; Toriello et al., 2021)

Sleep is often associated with cluster headache. According to a polysomnographic study, patients with cluster headache have a lower proportion of sleep stage R (previously referred to as REM sleep), and a long latency for sleep stage R (Barloese, Jennum et al., 2015). Another study also demonstrated longer sleep latency and lower sleep efficiency in patients with cluster headache compared to healthy controls (Lund et al., 2019). Furthermore, numerous sleep disorders have been linked to cluster headaches (Weintraub, 2003). Cluster headaches have been treated with melatonin and other treatments that affect circadian rhythm. Cluster headache patients may develop obstructive sleep apnea (Weintraub, 2003). A study involving mice demonstrated that natural sleep is linked to a 60% increase in the interstitial space of the brain. Because of the high hydraulic resistance in the brain interstitial space during alertness, sleep can increase the convective flow of CSF plus ISF; therefore, there is an increase in the efficiency of the glymphatic system during sleep (Xie et al., 2013). Several patients with cluster headache have attacks that wake them up, but they usually have sleep difficulties before the attacks, and sleep disorders are common concomitant diseases for patients with cluster headache (Martins, 2015). In addition, cluster headache frequently begins during sleep (Barloese, Lund et al., 2015; Barloese, Jennum et al., 2015). Thus, sleep disturbance in patients with cluster headache is thought to diminish the efficacy of glymphatic system function by lowering the flow of CSF and ISF.

Aging is closely related to sleep; therefore, sleep quality deteriorates with age. With aging, insomnia becomes more common, the total duration of sleep becomes shortened, and sleep becomes more disrupted. More importantly, older people rarely reach the deep sleep stage N (previously referred to as non‐REM sleep). In adults aged >60 years, the sleep stage N is predominantly shallow, consisting of the shallower sleep stage 1 or 2 (Landolt & Borbély, 2001). As a result, the older brain spends less time in sleep stage 3, potentially resulting in a catastrophic decrease in brain waste clearance, as the efficacy of glymphatic fluid transport is directly proportional to the prevalence of slow‐wave activity (Hablitz et al., 2019). These sleep abnormalities in patients with cluster headache are comparable to those observed with age, and this is thought to represent a mechanism of glymphatic system dysfunction. The CSF formation rate and turnover in the rat brain decrease with age, which is consistent with evidence showing that glymphatic clearance decreases with age (Kress et al., 2014). In this study, there was a negative correlation between the DTI‐ALPS index and age in patients with cluster headache, which is consistent with the results of previous animal experiments.

The limitations of this study are as follows. First, this was a retrospective study. The study confirmed that alterations in glymphatic system function were identified in patients with cluster headache; however, it is not clear whether this change is the result or cause of cluster headache. Second, there was a limited number of participants; therefore, it is difficult to generalize the results. However, cluster headache is not common, and we only enrolled newly diagnosed patients with cluster headache to exclude medication effects. Further studies with large sample size are needed to confirm our findings. Third, various factors can influence the DTI‐ALPS index, including the imaging plane, head position, and motion‐proving gradients (Taoka et al., 2021). However, a previous study found that the results of the DTI‐ALPS method were significantly related to glymphatic system function as measured by MRI after intrathecal administration of GBCA (Zhang et al., 2021), and another recent study successfully demonstrated good test‐retest reproducibility and robustness (Taoka et al., 2021). Despite these limitations, this study is notable because, to the best of our knowledge, no studies have investigated the link between the glymphatic system and cluster headache.

## 5 CONCLUSION

Patients with cluster headache had a lower DTI‐ALPS index than the healthy controls. This might indicate glymphatic system dysfunction in patients with cluster headache. Further research is required to determine whether glymphatic system dysfunction is related to the pathophysiology of cluster headache.

## CONFLICT OF INTEREST

The authors declare no conflict of interest.

## AUTHOR CONTRIBUTIONS

*Conception and design*: Jinseung Kim and Kang Min Park. *Acquisition of data, analysis and interpretation of data*: Dong Ah Lee, Ho‐Joon Lee, Bong Soo Park, Junghae Ko, S Park, Yoo Jin Lee, Il Hwan Kim, Jin Han Park, and Kang Min Park. *Drafting the manuscript or revising*: Jinseung Kim and KM Park. Final approval: Kang Min Park.

### PEER REVIEW

The peer review history for this article is available at https://publons.com/publon/10.1002/brb3.2631

## Data Availability

The data that support the findings of this study are available from the corresponding author upon reasonable request.

## References

[brb32631-bib-0001] Barloese, M. , Jennum, P. , Lund, N. , & Jensen, R. (2015). Sleep in cluster headache—Beyond a temporal rapid eye movement relationship? European Journal of Neurology, 22(4), 656–e640. 25557272 10.1111/ene.12623

[brb32631-bib-0002] Barloese, M. , Lund, N. , Petersen, A. , Rasmussen, M. , Jennum, P. , & Jensen, R. (2015). Sleep and chronobiology in cluster headache. Cephalalgia, 35(11), 969–978. 25573893 10.1177/0333102414564892

[brb32631-bib-0003] Debaker, C. , Djemai, B. , Ciobanu, L. , Tsurugizawa, T. , & Le Bihan, D. (2020). Diffusion MRI reveals in vivo and non‐invasively changes in astrocyte function induced by an aquaporin‐4 inhibitor. PLoS One, 15(5), e0229702. 32413082 10.1371/journal.pone.0229702 PMC7228049

[brb32631-bib-0004] Edeklev, C. , Halvorsen, M. , Løvland, G. , Vatnehol, S. , Gjertsen, Ø. , Nedregaard, B. , Sletteberg, R. , Ringstad, G. , & Eide, P. (2019). Intrathecal use of gadobutrol for glymphatic MR imaging: Prospective safety study of 100 patients. American Journal of Neuroradiology, 40(8), 1257–1264. 31320462 10.3174/ajnr.A6136 PMC7048483

[brb32631-bib-0005] Favoni, V. , Sambati, L. , Oppi, F. , Stanzani Maserati, M. , Cevoli, S. , & Pierangeli, G. (2018). Recurrent reversible cognitive impairment in a cluster headache patient: A case report. Headache: The Journal of Head and Face Pain, 58(9), 1472–1474. 10.1111/head.13393 30178567

[brb32631-bib-0006] Gaberel, T. , Gakuba, C. , Goulay, R. , De Lizarrondo, S. M. , Hanouz, J.‐L. , Emery, E. , Touze, E. , Vivien, D. , & Gauberti, M. (2014). Impaired glymphatic perfusion after strokes revealed by contrast‐enhanced MRI: A new target for fibrinolysis? Stroke; A Journal of Cerebral Circulation, 45(10), 3092–3096. 10.1161/STROKEAHA.114.006617 25190438

[brb32631-bib-0007] Goulay, R. , Flament, J. , Gauberti, M. , Naveau, M. , Pasquet, N. , Gakuba, C. , Emery, E. , Hantraye, P. , Vivien, D. , Aron‐Badin, R. , & Gaberel, T. (2017). Subarachnoid hemorrhage severely impairs brain parenchymal cerebrospinal fluid circulation in nonhuman primate. Stroke; A Journal of Cerebral Circulation, 48(8), 2301–2305. 10.1161/STROKEAHA.117.017014 28526764

[brb32631-bib-0008] Hablitz, L. M. , Vinitsky, H. S. , Sun, Q. , Stæger, F. F. , Sigurdsson, B. , Mortensen, K. N. , Lilius, T. O. , & Nedergaard, M. (2019). Increased glymphatic influx is correlated with high EEG delta power and low heart rate in mice under anesthesia. Science Advances, 5(2), eaav5447. 30820460 10.1126/sciadv.aav5447 PMC6392807

[brb32631-bib-0009] Headache Classification Committee of the International Headache Society (IHS) . (2018). Headache Classification Committee of the International Headache Society (IHS) The International Classification of Headache Disorders, 3rd edition. Cephalalgia, 38(1), 1–211. 10.1177/0333102417738202 29368949

[brb32631-bib-0010] Iliff, J. J. , Chen, M. J. , Plog, B. A. , Zeppenfeld, D. M. , Soltero, M. , Yang, L. , Singh, I. , Deane, R. , & Nedergaard, M. (2014). Impairment of glymphatic pathway function promotes tau pathology after traumatic brain injury. Journal of Neuroscience, 34(49), 16180–16193. 25471560 10.1523/JNEUROSCI.3020-14.2014 PMC4252540

[brb32631-bib-0011] Iliff, J. J. , Lee, H. , Yu, M. , Feng, T. , Logan, J. , Nedergaard, M. , & Benveniste, H. (2013). Brain‐wide pathway for waste clearance captured by contrast‐enhanced MRI. The Journal of Clinical Investigation, 123(3), 1299–1309. 23434588 10.1172/JCI67677 PMC3582150

[brb32631-bib-0012] Jang, H. , Lee, J. Y. , Lee, K. I. , & Park, K. M. (2017). Are there differences in brain morphology according to handedness? Brain and Behavior, 7(7), e00730. 10.1002/brb3.730 28729936 PMC5516604

[brb32631-bib-0013] Jensen, R. , Lyngberg, A. , & Jensen, R. (2007). Burden of cluster headache. Cephalalgia, 27(6), 535–541. 17459083 10.1111/j.1468-2982.2007.01330.x

[brb32631-bib-0014] Jiang, Q. , Zhang, L. , Ding, G. , Davoodi‐Bojd, E. , Li, Q. , Li, L. , Sadry, N., Nedergaard, M., Chopp, M., & Zhang, Z. (2017). Impairment of the glymphatic system after diabetes. Journal of Cerebral Blood Flow & Metabolism, 37(4), 1326–1337. 27306755 10.1177/0271678X16654702 PMC5453454

[brb32631-bib-0015] Kress, B. T. , Iliff, J. J. , Xia, M. , Wang, M. , Wei, H. S. , Zeppenfeld, D. , Xie, L. , Kang, H. , Xu, Q. , Liew, J. A. , Plog, B. A. , Ding, F. , Deane, R. , & Nedergaard, M. (2014). Impairment of paravascular clearance pathways in the aging brain. Annals of Neurology, 76(6), 845–861. 25204284 10.1002/ana.24271 PMC4245362

[brb32631-bib-0016] Landolt, H.‐P. , & Borbély, A. A. (2001). Age‐dependent changes in sleep EEG topography. Clinical Neurophysiology, 112(2), 369–377. 11165543 10.1016/s1388-2457(00)00542-3

[brb32631-bib-0017] Lee, H. J. , Lee, D. A. , Shin, K. J. , & Park, K. M. (2022). Glymphatic system dysfunction in patients with juvenile myoclonic epilepsy. Journal of Neurology, 269(4), 2133–2139. 10.1007/s00415-021-10799-w 34510256

[brb32631-bib-0018] Lund, N. L. T. , Snoer, A. H. , Petersen, A. S. , Beske, R. P. , Jennum, P. J. , Jensen, R. H. , & Barloese, M. C. J. (2019). Disturbed sleep in cluster headache is not the result of transient processes associated with the cluster period. European Journal of Neurology, 26(2), 290–298. 10.1111/ene.13820 30300455

[brb32631-bib-0019] Martins, I. P. (2015). Cyclic nocturnal awakening: A warning sign of a cluster bout. Cephalalgia, 35(4), 363–365. 24958682 10.1177/0333102414540057

[brb32631-bib-0020] Messlinger, K. (2018). The big CGRP flood—sources, sinks and signalling sites in the trigeminovascular system. Journal of Headache and Pain, 19(1), 22. 10.1186/s10194-018-0848-0 29532195 PMC5847494

[brb32631-bib-0021] Mestre, H. , Tithof, J. , Du, T. , Song, W. , Peng, W. , Sweeney, A. M. , Olveda, G. , Thomas, J. H. , Nedergaard, M. , & Kelley, D. H. (2018). Flow of cerebrospinal fluid is driven by arterial pulsations and is reduced in hypertension. Nature Communications, 9(1), 1–9. 10.1038/s41467-018-07318-3 PMC6242982 30451853

[brb32631-bib-0022] Nedergaard, M. , & Goldman, S. A. (2020). Glymphatic failure as a final common pathway to dementia. Science, 370(6512), 50–56. 10.1126/science.abb8739 33004510 PMC8186542

[brb32631-bib-0023] Peng, W. , Achariyar, T. M. , Li, B. , Liao, Y. , Mestre, H. , Hitomi, E. , Regan, S. , Kasper, T. , Peng, S. , Ding, F. , Benveniste, H. , Nedergaard, M. , & Deane, R. (2016). Suppression of glymphatic fluid transport in a mouse model of Alzheimer's disease. Neurobiology of Disease, 93, 215–225. 27234656 10.1016/j.nbd.2016.05.015 PMC4980916

[brb32631-bib-0024] Plog, B. A. , Dashnaw, M. L. , Hitomi, E. , Peng, W. , Liao, Y. , Lou, N. , Deane, R. , & Nedergaard, M. (2015). Biomarkers of traumatic injury are transported from brain to blood via the glymphatic system. Journal of Neuroscience, 35(2), 518–526. 25589747 10.1523/JNEUROSCI.3742-14.2015 PMC4293408

[brb32631-bib-0025] Ringstad, G. , Valnes, L. M. , Dale, A. M. , Pripp, A. H. , Vatnehol, S.‐A. S. , Emblem, K. E. , Mardal, K. A. , & Eide, P. K. (2018). Brain‐wide glymphatic enhancement and clearance in humans assessed with MRI. JCI Insight, 3(13), e121537. 10.1172/jci.insight.121537 PMC6124518 29997300

[brb32631-bib-0026] Rubinsztein, D. C. (2006). The roles of intracellular protein‐degradation pathways in neurodegeneration. Nature, 443(7113), 780–786. 17051204 10.1038/nature05291

[brb32631-bib-0027] Schain, A. J. , Melo‐Carrillo, A. , Strassman, A. M. , & Burstein, R. (2017). Cortical spreading depression closes paravascular space and impairs glymphatic flow: Implications for migraine headache. Journal of Neuroscience, 37(11), 2904–2915. 10.1523/JNEUROSCI.3390-16.2017 28193695 PMC5354333

[brb32631-bib-0028] Sjaastad, O. (1992). Cluster headache syndrome. Major Problems in Neurology, 42(7), 1438.

[brb32631-bib-0029] Headache Classification Committee of the International Headache Society (IHS) . (2013). The international classification of headache disorders, (beta version). Cephalalgia, 33(9), 629–808. 23771276 10.1177/0333102413485658

[brb32631-bib-0030] Sun, B.‐L. , Wang, L. H. , Yang, T. , Sun, J. Y. , Mao, L. L. , Yang, M. F. , Yuan, H. , Colvin, R. A. , & Yang, X. Y. (2018). Lymphatic drainage system of the brain: A novel target for intervention of neurological diseases. Progress in Neurobiology, 163, 118–143. 28903061 10.1016/j.pneurobio.2017.08.007

[brb32631-bib-0031] Taoka, T. , Ito, R. , Nakamichi, R. , Kamagata, K. , Sakai, M. , Kawai, H. , Nakane, T. , Abe, T. , Ichikawa, K. , Kikuta, J. , Aoki, S. , & Naganawa, S. (2022). Reproducibility of diffusion tensor image analysis along the perivascular space (DTI‐ALPS) for evaluating interstitial fluid diffusivity and glymphatic function: CHanges in Alps index on Multiple conditiON acquIsition eXperiment (CHAMONIX) study. Japanese Journal of Radiology, 40(2), 147–158. 34390452 10.1007/s11604-021-01187-5 PMC8803717

[brb32631-bib-0032] Taoka, T. , Masutani, Y. , Kawai, H. , Nakane, T. , Matsuoka, K. , Yasuno, F. , Kishimoto, T. , & Naganawa, S. (2017). Evaluation of glymphatic system activity with the diffusion MR technique: Diffusion tensor image analysis along the perivascular space (DTI‐ALPS) in Alzheimer's disease cases. Japanese Journal of Radiology, 35(4), 172–178. 10.1007/s11604-017-0617-z 28197821

[brb32631-bib-0033] Toriello, M. , Gonzalez‐Quintanilla, V. , Perez‐Pereda, S. , Fontanillas, N. , & Pascual, J. (2021). The potential role of the glymphatic system in headache disorders. Pain Medicine, 22(12), 3098–3100. 10.1093/pm/pnab137 33839781

[brb32631-bib-0034] Vgontzas, A. , & Pavlovic, J. M. (2018). Sleep disorders and migraine: review of literature and potential pathophysiology mechanisms. Headache, 58(7), 1030–1039. 10.1111/head.13358 30091160 PMC6527324

[brb32631-bib-0035] Wang, M. , Ding, F. , Deng, S. , Guo, X. , Wang, W. , Iliff, J. J. , & Nedergaard, M. (2017). Focal solute trapping and global glymphatic pathway impairment in a murine model of multiple microinfarcts. Journal of Neuroscience, 37(11), 2870–2877. 28188218 10.1523/JNEUROSCI.2112-16.2017 PMC5354332

[brb32631-bib-0036] Weintraub, J. R. (2003). Cluster headaches and sleep disorders. Current Pain and Headache Reports, 7(2), 150–156. 10.1007/s11916-003-0026-0 12628058

[brb32631-bib-0037] Xie, L. , Kang, H. , Xu, Q. , Chen, M. J. , Liao, Y. , Thiyagarajan, M. , O'Donnell, J. , Christensen, D. J. , Nicholson, C. , Iliff, J. J. , Takano, T. , Deane, R. , & Nedergaard, M. (2013). Sleep drives metabolite clearance from the adult brain. Science, 342(6156), 373–377. 24136970 10.1126/science.1241224 PMC3880190

[brb32631-bib-0038] Yokota, H. , Vijayasarathi, A. , Cekic, M. , Hirata, Y. , Linetsky, M. , Ho, M. , Kim, W. , & Salamon, N. (2019). Diagnostic performance of glymphatic system evaluation using diffusion tensor imaging in idiopathic normal pressure hydrocephalus and mimickers. Current Gerontology And Geriatrics Research, 2019, 5675014. 31320896 10.1155/2019/5675014 PMC6609364

[brb32631-bib-0039] Zhang, W. , Zhou, Y. , Wang, J. , Gong, X. , Chen, Z. , Zhang, X. , Cai, J. , Chen, S. , Fang, L. , Sun, J. , & Lou, M. (2021). Glymphatic clearance function in patients with cerebral small vessel disease. Neuroimage, 238, 118257. 34118396 10.1016/j.neuroimage.2021.118257

